# Brucellosis Ontology (IDOBRU) as an extension of the Infectious Disease Ontology

**DOI:** 10.1186/2041-1480-2-9

**Published:** 2011-10-31

**Authors:** Yu Lin, Zuoshuang Xiang, Yongqun He

**Affiliations:** 1 Unit for Laboratory Animal Medicine, University of Michigan Medical School, Ann Arbor, MI 48109, USA; 2 Department of Microbiology and Immunology, University of Michigan Medical School, Ann Arbor, MI 48109, USA; 3 Center for Computational Medicine and Bioinformatics, University of Michigan Medical School, Ann Arbor, MI 48109, USA

## Abstract

**Background:**

Caused by intracellular Gram-negative bacteria *Brucella *spp., brucellosis is the most common bacterial zoonotic disease. Extensive studies in brucellosis have yielded a large number of publications and data covering various topics ranging from basic *Brucella *genetic study to vaccine clinical trials. To support data interoperability and reasoning, a community-based brucellosis-specific biomedical ontology is needed.

**Results:**

The Brucellosis Ontology (IDOBRU: http://sourceforge.net/projects/idobru), a biomedical ontology in the brucellosis domain, is an extension ontology of the core Infectious Disease Ontology (IDO-core) and follows OBO Foundry principles. Currently IDOBRU contains 1503 ontology terms, which includes 739 *Brucella*-specific terms, 414 IDO-core terms, and 350 terms imported from 10 existing ontologies. IDOBRU has been used to model different aspects of brucellosis, including host infection, zoonotic disease transmission, symptoms, virulence factors and pathogenesis, diagnosis, intentional release, vaccine prevention, and treatment. Case studies are typically used in our IDOBRU modeling. For example, diurnal temperature variation in *Brucella *patients, a *Brucella*-specific PCR method, and a WHO-recommended brucellosis treatment were selected as use cases to model brucellosis symptom, diagnosis, and treatment, respectively. Developed using OWL, IDOBRU supports OWL-based ontological reasoning. For example, by performing a Description Logic (DL) query in the OWL editor Protégé 4 or a SPARQL query in an IDOBRU SPARQL server, a check of *Brucella *virulence factors showed that eight of them are known protective antigens based on the biological knowledge captured within the ontology.

**Conclusions:**

IDOBRU is the first reported bacterial infectious disease ontology developed to represent different disease aspects in a formal logical format. It serves as a brucellosis knowledgebase and supports brucellosis data integration and automated reasoning.

## Background

Brucellosis is a zoonotic infectious disease caused by intracellular Gram-negative bacteria *Brucella *spp. Since its initial isolation from tissue of a deceased patient by Dr. David Bruce in 1887 [1], *Brucella *has been found in many animals, including cattle, pigs, goat, sheep, dogs, fish and so on. Human brucellosis remains the most common zoonotic disease worldwide, with more than 500, 000 new cases reported annually [2]. The variety of its clinical manifestations makes diagnosis and treatment difficult. Currently there is no available human brucellosis vaccine. As a select agent categorized by the USA Centers for Disease Control and Prevention (CDC), aerosolized *Brucella *can also be used for bioterrorism [3].

Many online resources for *Brucella *information are available. For example, *Brucella *Bioinformatics Portal (BBP) is a web portal that allows users to search and analyze individual *Brucella *genes and link to more than 20 existing databases and analysis programs [4]. PATRIC Pathosystems resource supports browsing, visualization, and detailed analyses of *Brucella *genomes [5]. VIOLIN vaccine database and analysis system stores information of licensed *Brucella *vaccines and vaccine candidates [6]. The *Brucella *data collected in these resources has been widely used for various purposes by researchers around the world. However, the following bottlenecks prevent advanced data exchange and integration among these online resources: (1) frequent use of different terminologies for the same concepts, (2) a lack of logical and machine-readable relations among different terms, and (3) a lack of machine-readable and community-supported data exchange format (*e.g*., the OWL format) for representation of the *Brucella *data. These obstacles prevent computer-assisted automated reasoning.

Biomedical ontologies are sets of hierarchical terms and relations that represent entities in the biomedical science and show how these entities relate to each other. To support automated reasoning, ontological terms are often expressed in formal logic, together with documentation and definitions [7-9]. Biomedical ontologies play important roles in areas such as knowledge management (including data indexing and information retrieval); data integration, exchange and semantic interoperability; and decision support and reasoning [10]. To facilitate translational medical research in infectious diseases, an Infectious Disease Ontology (IDO) Consortium http://www.infectiousdiseaseontology.org/ has been established. IDO is aimed to develop a suite of interoperable ontologies that jointly cover the entire infectious disease domain, spanning infectious disease specialties and the clinical care, public health, and biomedical research domains. The IDO suite of ontologies are developed using a core-extension approach in which disease- or pathogen-specific ontologies are developed as extension ontologies from a common IDO core [11]. The IDO-core ontology provides coverage of those entities relevant to infectious diseases generally, including terms such as host, pathogen, focal infection, and herd immunity. Meanwhile, the IDO extensions cover entities relevant to specific disease or pathogen types. The IDO-core was developed within the framework of the Basic Formal Ontology (BFO) [9] and the Ontology of General Medical Sciences (OGMS) [12]. The IDO-core terms have simple and formal natural language definitions as well as formal logical expressions stated in terms of relations from the OBO relation ontology and terms from IDO or other OBO ontologies, such as the Gene Ontology (GO) [7]. Specific IDO extension ontologies (*e.g*., Brucellosis Ontology, Influenza Ontology [13], and Malaria Ontology [14]) are maintained by experts from specific infectious disease areas. Different IDO extension ontologies are developed from the core in a coordinated fashion that ensures interoperability among all IDO extension ontologies.

In this paper, we report our development of a Brucellosis Ontology (IDOBRU) as an IDO-core extension ontology that targets the brucellosis domain.

## Results

In what follows, *italics *are used to refer to a specific term within IDOBRU where appropriate. Prefix of an existing ontology is listed before one term, in such a form as *OBI:data item*. By default, the IDOBRU terms in this paper have no prefix. Additional file 1 provides detailed information of all ontology terms and relations used in this manuscript.

### IDOBRU general design and introduction

IDOBRU is an ontology that covers and crosses the biomedical domains of clinical care, public health and biomedical research in the specific brucellosis field. IDOBRU contains the vocabulary and terms from seven major aspects: host infection and zoonotic disease transmission, symptoms, virulence factors and pathogenesis, diagnosis, intentional release, vaccine prevention, and treatment. The goal of IDOBRU is to establish a knowledgebase of *Brucella *and brucellosis. The targeted users of IDOBRU include brucellosis researchers, microbiologists, bioinformaticians, clinicians, governments and related decision makers.

**Figure 1:**
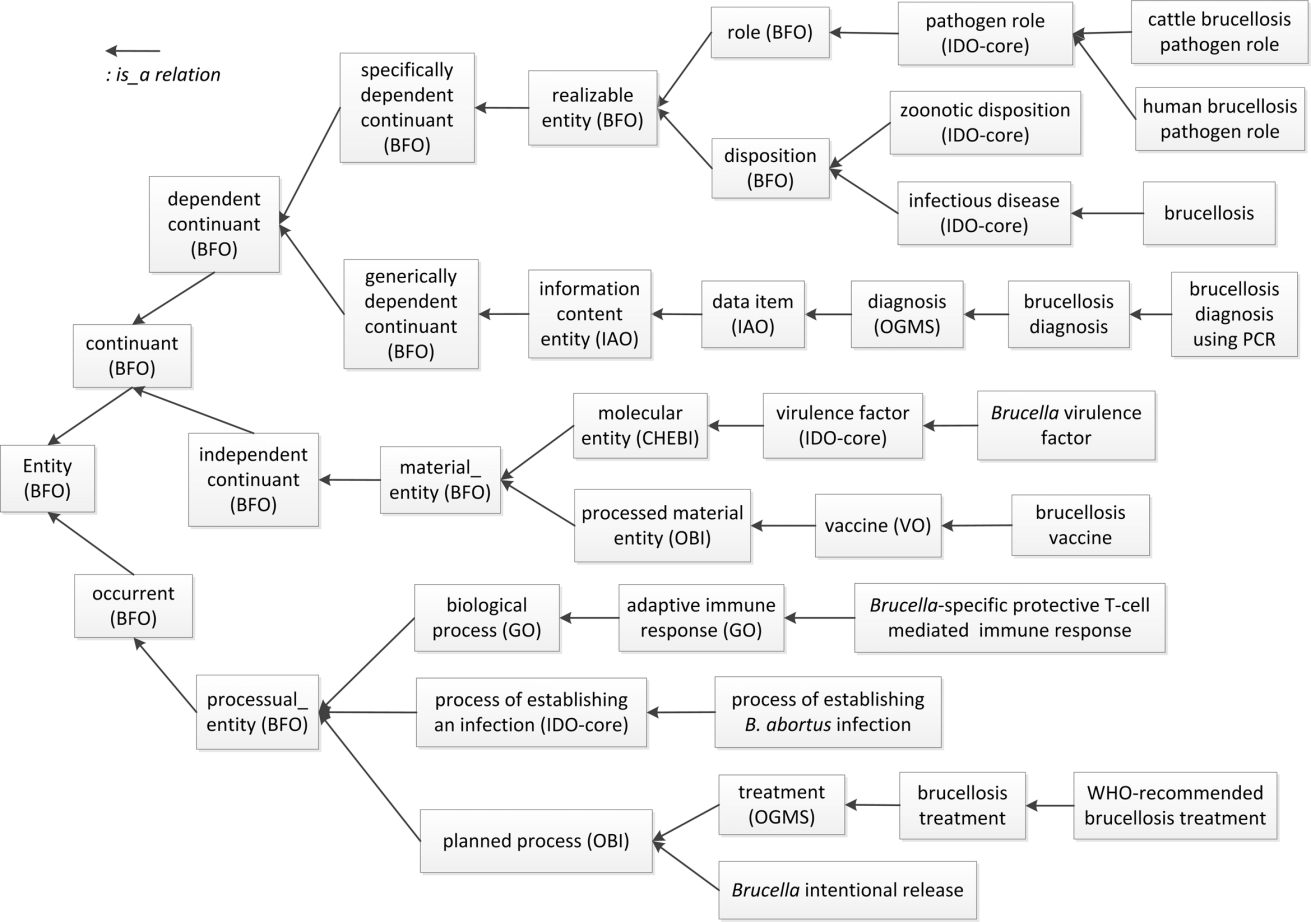
**The major architecture of IDOBRU**. Key top level ontology terms in IDOBRU are included. Those terms that do not include any ontology acronyms are generated in the IDOBRU ontology. Each arrow sign "←" represents an *is_a *relation where a right side class term is a subclass of the term at the left side of the arrow.

The current IDOBRU version "Arbor Release" (version number 1.1.41 released on October 28, 2011) contains a total of 1503 terms, including 1469 classes, 26 object properties, and 8 datatype properties. Among them, 414 terms are IDO-core-specific terms. In total, IDOBRU includes 739 IDOBRU-specific terms, including 726 classes, 5 object properties and 8 datatype properties. Following the principles of OBO Foundry, IDOBRU has reused or adopted external ontologies that are OBO Foundry ontologies and candidate ontologies. IDOBRU fully imports the whole BFO, RO and IDO-core. Using the OntoFox [15] software program, IDOBRU imports external terms from eight other existing ontologies and resources: Chemical Entities of Biological Interest (CHEBI) [16], Gene Ontology [7], Information Artifact Ontology (IAO) [17], NCBI Taxonomy database [18], Ontology for Biomedical Investigations (OBI) [19], Ontology for General Medical Science (OGMS) [12], Protein Ontology [20], and Vaccine Ontology (VO) [21] (Table 1). In total, IDOBRU has imported 764 terms from 11 external ontologies. Figure 1 shows the major architecture of IDOBRU that includes key top-level terms in IDOBRU. As shown in this figure, all brucellosis-specific terms are subclasses of terms from higher level ontologies including the IDO-core, OGMS, OBI, VO, and GO.

**Table 1:** IDOBRU specific terms and terms imported to IDOBRU from 11 source ontologies

#	Ontology	Classes	Object Properties	Datatype Properties	*Total*
1	IDOBRU (Brucellosis Ontology) specific	726	5	8	739

*Imported ontologies as whole*					

1	BFO (Basic Formal Ontology)	39	0	0	39

2	RO (Relation Ontology)	0	9	0	9

3	IDO (Infectious Disease Ontology)	411	3	0	414

4	OGMS (Ontology for General Medical Science)	82	0	0	82

*Imported from other external ontologies*					

1	OBI (Ontology for Biomedical Investigations)	12	7	0	19

2	NCBITaxon (NCBI Taxonomy)	103	0	0	103

3	IAO (Information Artifact Ontology)	7	2	0	9

4	VO (Vaccine Ontology) specific	68	0	0	68

5	CHEBI (Chemical Entities of Biological Interest)	9	0	0	11

6	GO (Gene Ontology)	9	0	0	9

7	PRO (Protein Ontology)	1	0	0	1

*Total*	12	1469	26	8	1503

According to an agreement between IDOBRU and the IDO-core development teams, a "hub-and-spoke" model is adopted for the development of the IDO-core and IDO extension ontologies. Specifically, the IDO-core acts as the hub and plays a role as the mediator for all extension ontologies, and infectious disease extension ontologies act as spokes. As a principal in OBO foundry ontologies, an identifier is always bipartite, in the form of *ID-space:Local-ID*, for example: IDO:0000001. Instead of using different ID-spaces and Local-IDs for individual IDO extension ontologies, every extension ontology uses the same ID-space: "IDO", as the IDO-core. For example, an ID used for an IDOBRU term is IDO:0100246, instead of IDOBRU:0000246. Unique ID blocks pre-assigned by the IDO-core team are used to differentiate specific extension ontologies. This strategy has many advantages: (1) to avoid duplicate terms and efforts; (2) to enforce the same best practice for ontology development across different extension ontologies; and (3) to encourage better collaboration and closer coordination between the IDO-core and IDO extension development teams. Meanwhile, each extension ontology is developed independently and maintains its own development repository site.

In the following sections, we will introduce IDOBRU representation of different aspects of brucellosis in details.

### Modeling brucellosis, host infections and pathogen transmission in IDOBRU

The genus *Brucella *is taxonomically placed in the alpha-2 subdivision of the class *NCBITaxon:Proteobacteria*. Traditionally there are eight species of *Brucella *based on the preferential host specificity: *B. melitensis *(goats), *B. abortus *(cattle), *B. suis *(swine), *B. canis *(dogs), *B. ovis *(sheep), *B. neotomae *(desert mice) [4], *B. cetaceae *(cetacean), and *B. pinnipediae *(seal) [22]. The first four species are pathogenic to humans in decreasing order of severity, making brucellosis a zoonotic disease.

**Figure 2:**
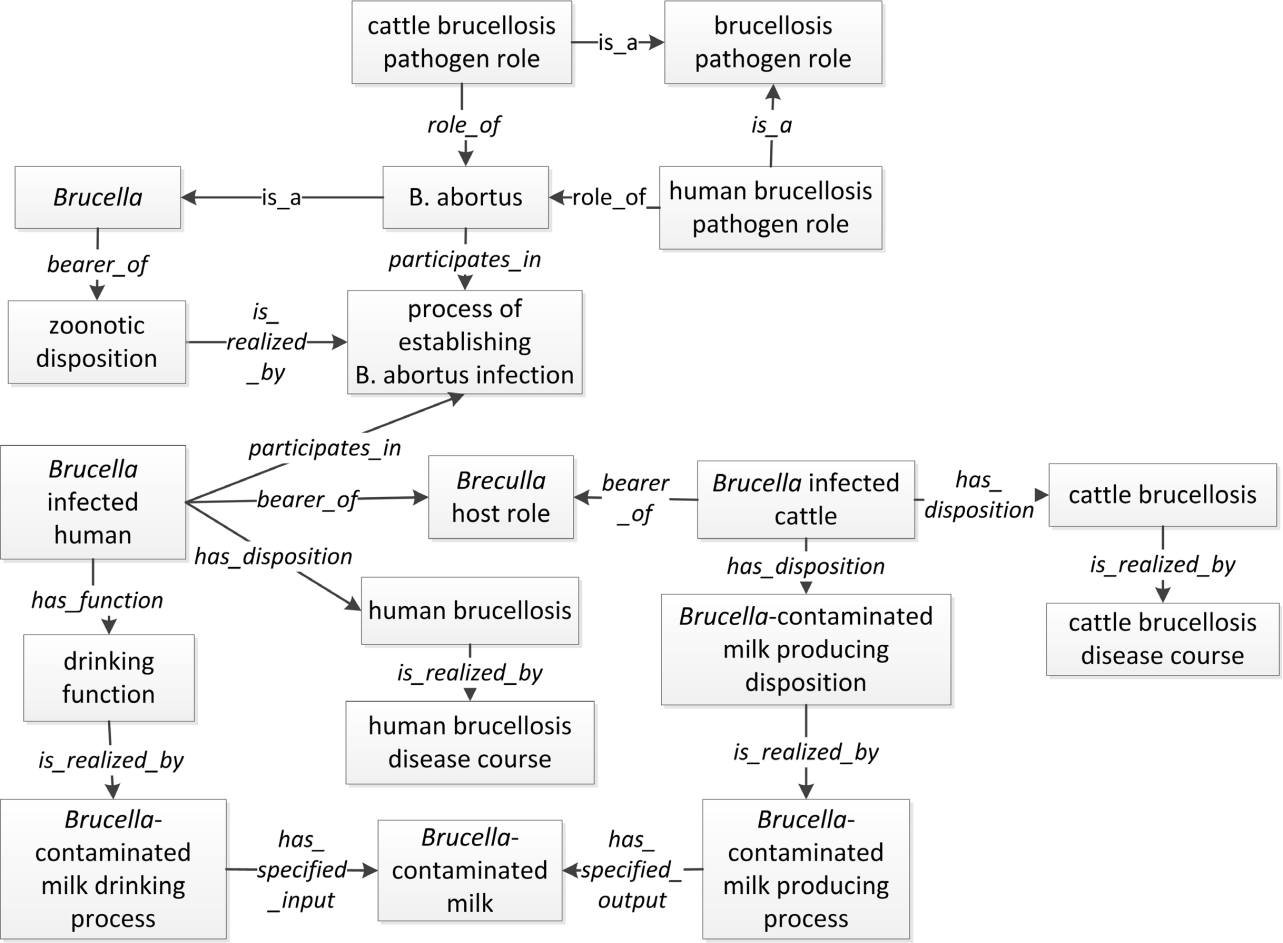
**IDOBRU modeling of human and cattle brucellosis due to zoonotic *Brucella *infections and transmission**. The terms inside boxes represent ontology classes. The terms in the middle of arrows are ontology relations.

The zoonosis feature of brucellosis is captured by the term of *IDO:zoonotic disposition*. In IDO, *zoonotic disposition *is defined as an infectious disposition that is the disposition to be transmitted from an infected, non-human host to a human host [23]. Therefore, in IDOBRU, *Brucella *is asserted as bearer of *IDO:zoonotic disposition *(Figure 2). For example, *B. abortus*, a subclass of *Brucella*, can infect both cattle and human. As subclasses of *brucellosis pathogen role*, both of the *human brucellosis pathogen role *and *cattle brucellosis pathogen role *are roles of *B. abortus*. During the *process of establishing B. abortus infection*, the *zoonotic disposition *of *Brucella *is realized. An infected human or cattle is the host of *Brucella *during the infection. Therefore, both *Brucella infected human *and *Brucella infected cattle *are the bearer of *Brucella host role. Brucella infected human (or cattle) *has disposition of *human (or cattle) brucellosis *, which is realized in the disease course of human (or cattle) brucellosis (Figure 2).

To model the complicated infection and animal-to-human transmission mechanisms in brucellosis, we used the following scenario as an example. A brucellosis human patient is infected via drinking the unpasteurized *Brucella*-contaminated milk produced by a *Brucella*-infected cow. The *Brucella-infected cow *produces milk that is contaminated with *Brucella *during the *Brucella-contaminated milk producing process*, where the *Brucella-contaminated milk producing disposition *is realized. The *Brucella-contaminated milk*, a specified output of this producing process, is drunk by the *Brucella infected human *when he/she realizes a *drinking function *(Figure 2).

### Modeling Brucellosis symptoms in IDOBRU

**Figure 3:**
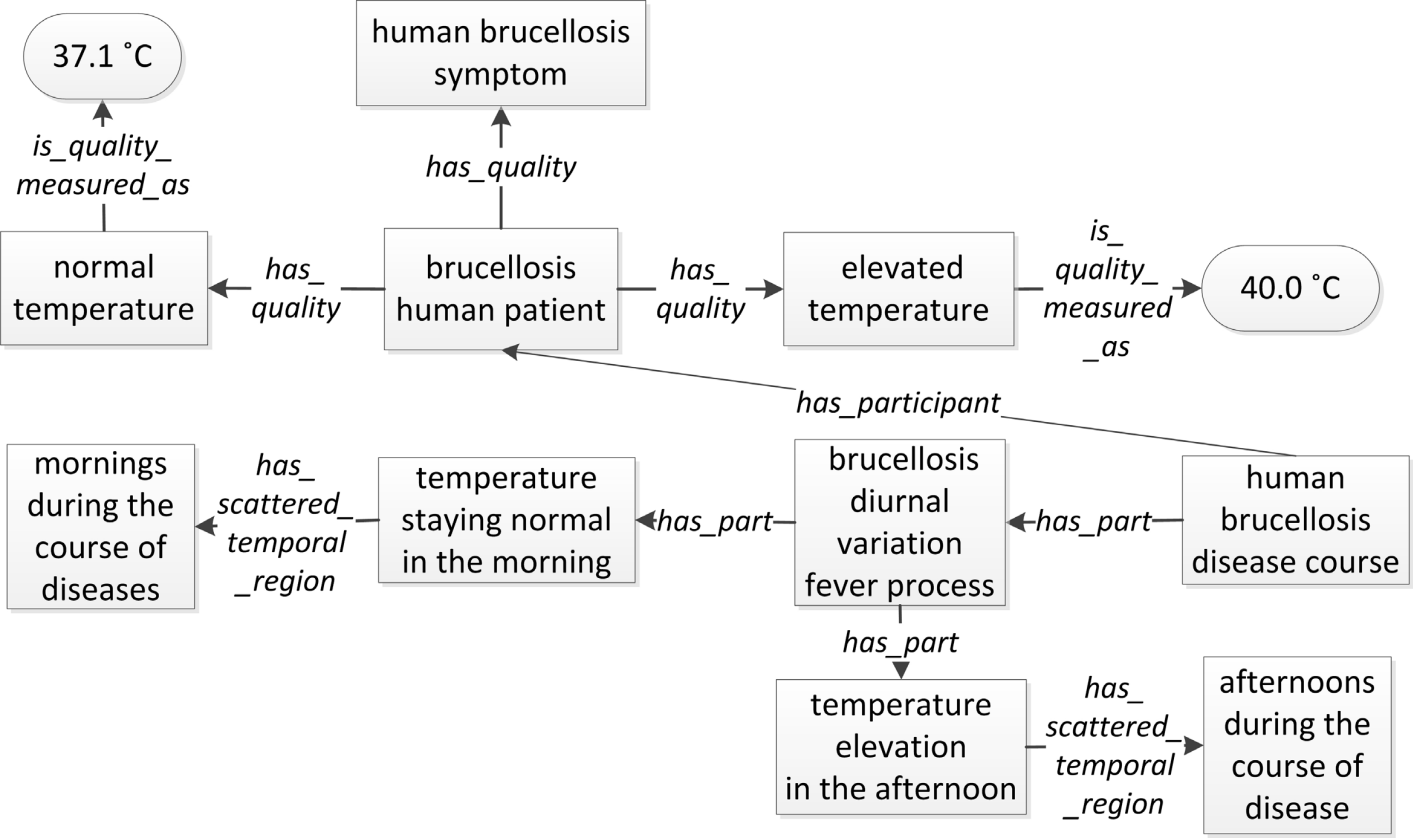
**IDOBRU modeling of *Brucella *undulant fever process**. The two temperatures (37.1°C and 40.0°C) inside oval boxes are instances. All other terms inside boxes represent ontology classes. The terms in the middle of arrows are ontology relations.

A symptom is a bodily feature that a patient observes. The *brucellosis symptom *is a subclass of *OGMS:symptom *[12]. Brucellosis usually causes abortion and sterility in non-human animals. Undulant fever, myalgia, and arthralgia of the large joints are the main symptoms in human brucellosis patients [24]. As a use case, the human undulant fever is modeled here (Figure 3). Undulant fever is a term with an ambiguous meaning. It has been used as an alternative synonym for human brucellosis, as well as a symptom of human brucellosis. In IDOBRU's point of view, the reality of the undulant fever is based on the fact that during the course of the disease, brucellosis patient's temperature shows diurnal variation [25]. As a scenario of the temperature diurnal variation process, a brucellosis human patient has a quality of *elevated temperature *measured as *40°C *in the afternoon, and then has a quality of *normal temperature *measured as *37.1°C *in the morning. Both *temperature elevation in the afternoon *and *temperature staying normal in the morning *are parts of the *process of diurnal variation of temperature*, which is a part of the *human brucellosis disease course*. The *human brucellosis symptom *is a quality of *brucellosis human patient*. At the different time points, the human patient has different temperature qualities as measured with different temperature numbers. It is noted that specific temperatures in different time points fall into instance levels of our ontology rather than class levels of entity. However, the *elevated temperature *and *normal temperature *without temporal part are considered as subclasses of *BFO:Quality *at the class level.

### Modeling *Brucella *virulence factors and pathogenesis in IDOBRU

**Figure 4:**
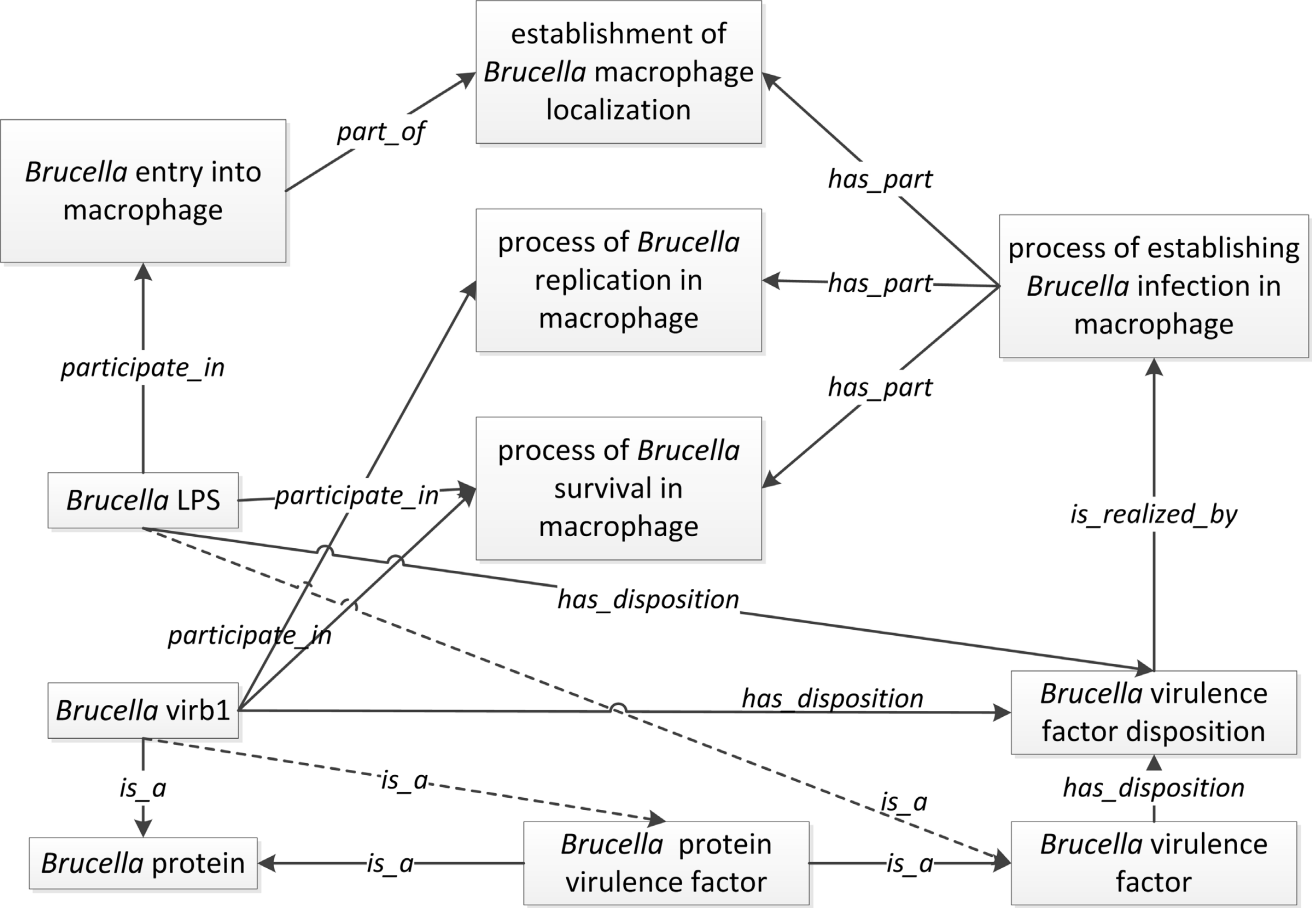
**IDOBRU modeling of *Brucella *virulence factors and pathogenesis**. The terms inside boxes represent ontology classes. The terms in the middle of arrows are ontology relations.

*Brucella *doesn't bear the classic bacterial virulence factors as capsules, secreted proteases, exotoxins, endotoxins, etc. As an intracellular bacterium, *Brucella *virulence relies on its ability to survive and replicate in the vacuolar compartments of macrophages. The interaction between *Brucella *and macrophages is critical for the establishment of a chronic infection [26,27]. Three processes are critically important in order for *Brucella *to establish a successful infection: (a) the entry into host cell, (b) the survival and (c) the replication within membrane-bound compartments in host cells, particularly macrophages. The type IV secretion system encoded by *Brucella virB *operon is required for the survival of *Brucella *inside replicative phagosomes in macrophages [28]. *Brucella *lipopolysaccharide (LPS) of smooth virulent *Brucella *strains is required to prevent the phagosome-lysosome fusion in macrophages, allowing the survival and replication of *Brucella *inside macrophages [28,29]. *Brucella *VirB1 protein and *Brucella *LPS, the two important virulence factors, are used as examples for virulence factor modeling in IDOBRU (Figure 4).

In IDO-core, the terms related to virulence factor are *IDO: virulence factor *and *IDO:virulence factor disposition. IDO:virulence *factor is the bearer of *IDO: virulence factor disposition*. As an IDO extension, IDOBRU has *Brucella virulence factor *and *Brucella virulence factor disposition*. Using the *Brucella-*macrophage interaction as a scenario, the *Brucella virulence factor disposition *is realized in the *process of establishing Brucella infection in macrophages*. As a process entity, the *process of establishing Brucella infection in macrophages *has three partial processes: *establishment of Brucella intracellular localization, Brucella survival in macrophages*, and *Brucella replication in macrophages *(Figure 4). The 'part of' relation (*RO:part_of*) has been asserted between each of these partial processes and the *process of establishing Brucella infection in macrophage. Brucella VirB1*, a subtype of *Brucella protein*, has the disposition as a *Brucella virulence factor disposition *and participates in the processes of *Brucella survival in macrophages *and *Brucella replication in macrophages. Brucella LPS *participates in the processes of *Brucella replication in macrophages*, and *Brucella entry into macrophages*, which is a partial process of *establishment of Brucella intracellular localization*. In IDOBRU, the virulence factors can be different types of molecular material such as protein and lipopolysaccharide. We asserted a term *Brucella protein virulence factor *as a subtype of *Brucella virulence factor *in IDOBRU. The formal logic definitions of *Brucella virulence factor *and *Brucella protein virulence factor *have been given as such:

*Brucella *virulence factor ≡ *(part_of some Brucella) and (bearer_of some 'Brucella virulence factor disposition')*

*Brucella *protein virulence factor ≡ *'Brucella protein' and (bearer_of some 'Brucella virulence factor disposition')*

After reasoning, *Brucella *VirB1 has been computed as a subclass of *Brucella protein virulence factor*, and *Brucella LPS *as a subclass of *Brucella virulence factor*.

In total, 245 curated *Brucella *virulence factors have been imported into IDOBRU from a MySQL database of the *Brucella *Bioinformatics Portal (BBP) [4].

### Modeling Diagnosis of brucellosis in IDOBRU

Diagnosis of human brucellosis cannot be made solely on clinical grounds due to the wide variety of its clinical manifestations. It is essential to confirm the diagnosis using bacteriological and serological tests. Many assays (*e.g*., polymerase chain reaction or PCR, ELISA, and agglutination assays) have been used for diagnosis of brucellosis [25]. The use of PCR allows for rapid and accurate diagnosis, and it is a rapid method to confirm the infection of *Brucella *[30]. IDOBRU adopts the terminological framework of OGMS for a top level representation of diseases, such as disease causes and manifestations, diagnosis, and other disease interpretations used in the clinic. According to OGMS, *diagnosis *is defined as "a conclusion of an interpretive process that has as input a clinical picture of a given patient and as output an assertion" [12]. *Brucellosis diagnosis *is a subclass of OGMS:*diagnosis*. Based on different assays used in the diagnostic process, brucellosis diagnosis has many subclasses, *e.g*., *brucellosis diagnosis by PCR test, brucellosis diagnosis by ELISA*, and *brucellosis diagnosis by microscopy*.

**Figure 5:**
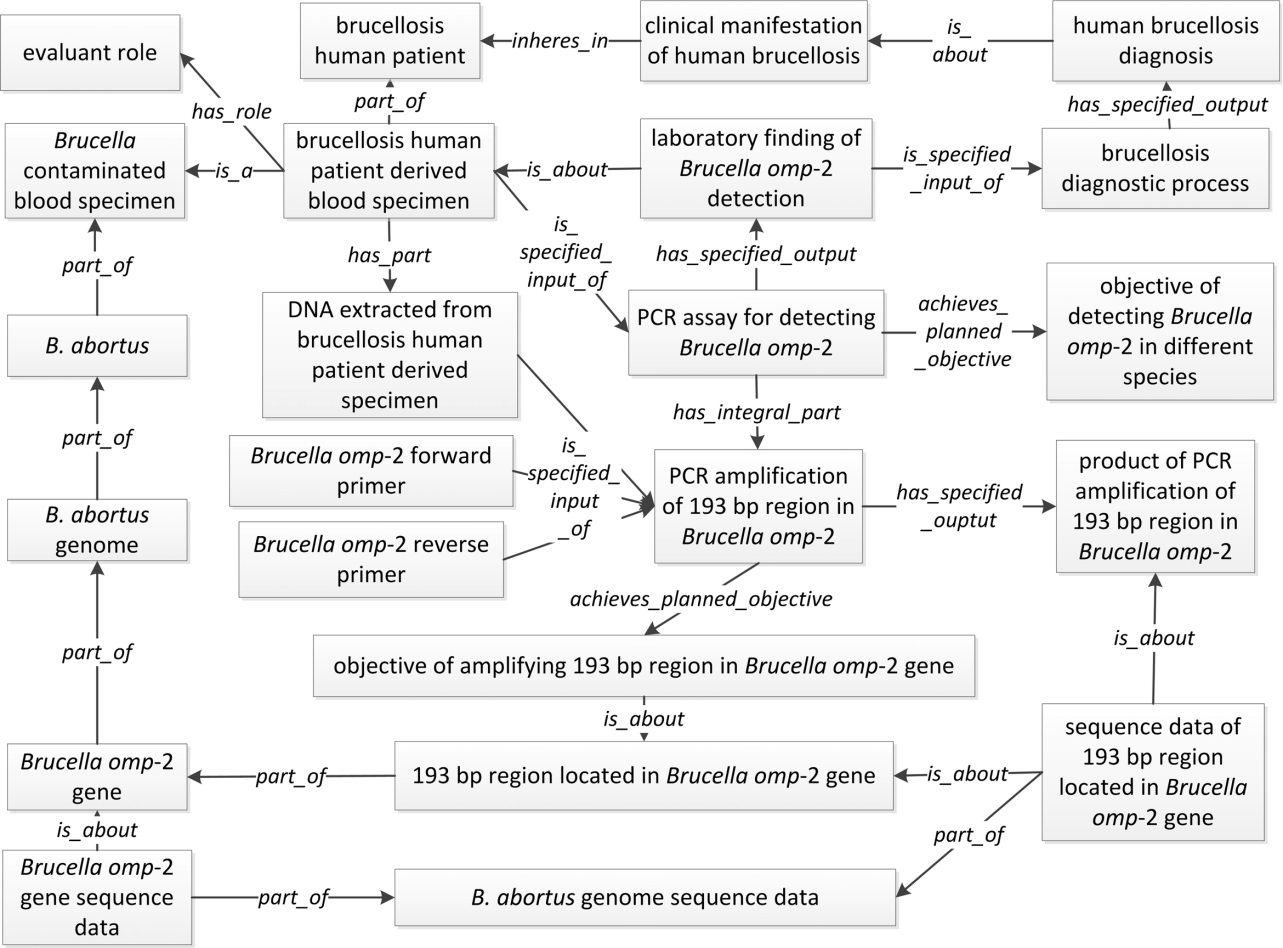
**IDOBRU modeling of brucellosis diagnosis using PCR test**. The terms inside boxes represent ontology classes. The terms in the middle of arrows are ontology relations.

PCR assays have been frequently used for diagnosis. A PCR assay is different from PCR amplification. The PCR amplification is a material transformation process with an output of amplified PCR product. A PCR assay is designed for specific purposes (*e.g*. detecting a target sequence for diagnosis) using PCR amplification. The PCR amplification is a part of a PCR assay. To model brucellosis diagnosis, a PCR assay used to test a *Brucella *gene *omp-2 *encoding for an outer membrane protein (OMP-2) from patient's blood sample is specifically studied [31] (Figure 5). First, the IDOBRU term *PCR assay for detecting Brucella omp-2 *was asserted as a subclass of *OGMS:laboratory test*. Using brucellosis patient derived specimen as specified input, the PCR assay will generate an output that is the *laboratory finding of Brucella omp-2 detection*. As a subclass of *IAO:information content entity*, this specific laboratory finding is about the patient derived specimen. The IAO relation *is_about *relates an information artifact to an entity. Secondly, this specific PCR assay has an integral part: *PCR to amplify 193 bp region in Brucella OMP-2 gene *[31]. *OBI:polymerase chain reaction *has been imported from OBI, and the *PCR amplification of 193 bp region in Brucella OMP-2 gene *is a subclass of *OBI:polymerase chain reaction *in IDOBRU. The inputs of the PCR amplification are: (a) *Brucella OMP-2 forward primer*, (b) *Brucella OMP-2 reverse primer*, and (c) DNA extracted from *Brucella *contaminated blood specimen. The output of this PCR amplification is the *product of PCR amplification of 169 bp region in Brucella OMP-2*. Finally, only a positive result obtained from the PCR assay would allow a doctor to draw a diagnostic conclusion of *Brucella *infection. Using the *laboratory finding of Brucella OMP-2 detection *as an input for the *brucellosis diagnostic process*, the output is *human brucellosis diagnosis*. This diagnosis thus *is_about *the *clinical manifestation of brucellosis *(Figure 5).

The primer sequence information and PCR product described in above PCR experiment are modeled as *primer sequence data *and *PCR product sequence data*, respectively. All of the sequence data of primers and PCR product are parts of *B. abortus *genome sequence data [31]. The relation *denotes *is defined in IAO that allows us to assert the relationship between digital data of a DNA sequence and its corresponding physical biological material sequence. In our case, PCR sequence data (a digital data item) *denotes *PCR product of the *Brucella *OMP-2 PCR amplification. A 'part-whole' relation is used in two different types of entities here: a) The material entity: *OMP-2 gene *as part of *B. abortus genome*, which is part of the *B. abortus *bacterial cell that exists in the blood specimen taken from the human patient. b) The information content entity: the *OMP-2 gene sequence data *as part of *B. abortus genome sequence data*.

### Modeling brucellosis epidemiology and intentional release in IDOBRU

Many factors can affect the prevalence of brucellosis in various species of livestock. Prevalence of brucellosis can vary according to climatic conditions, geography, species, sex, age and diagnostic tests applied [32]. Although it has been eradicated in many developed countries in Europe, Australia, Canada, Israel, Japan and New Zealand, brucellosis remains an uncontrolled problem in regions of high endemicity such as the Africa, Mediterranean, Middle East, parts of Asia and Latin America. Re-emergence of brucellosis is reported in Japan 1996, Bulgaria during 2005 to 2007 and FBH (Federation of Bosnia and Herzgovina) recently [25].

In IDOBRU, terms such as *eradication of brucellosis*, *Brucella accidental release, brucellosis endemic site*, *brucellosis free site*, *brucellosis non-endemic site*, *brucellosis surveillance *, and more others, have been used to capture the knowledge of epidemiology aspect of brucellosis. Since brucellosis is a zoonotic disease, the above classes have subclasses both in human brucellosis and non-human brucellosis.

*Brucella *can be intentionally used as a bioterrorism weapon as an incapacitating agent rather than a lethal agent. Although brucellosis causes low fatality rate, human brucellosis is a notoriously debilitating disease, and brucellosis patients require prolonged treatment [24]. *Brucella *organisms can be aerosolized and released in infectious doses, such as a sum of 10-500 virulent aerosol *Brucella *organisms. It is important to disinfect *Brucella *once its release is identified. Most commercial disinfectants are effective at killing or neutralizing *Brucella *organisms. Bleach (10%) has been used as an effective disinfectant to control the release of *Brucella*.

**Figure 6:**
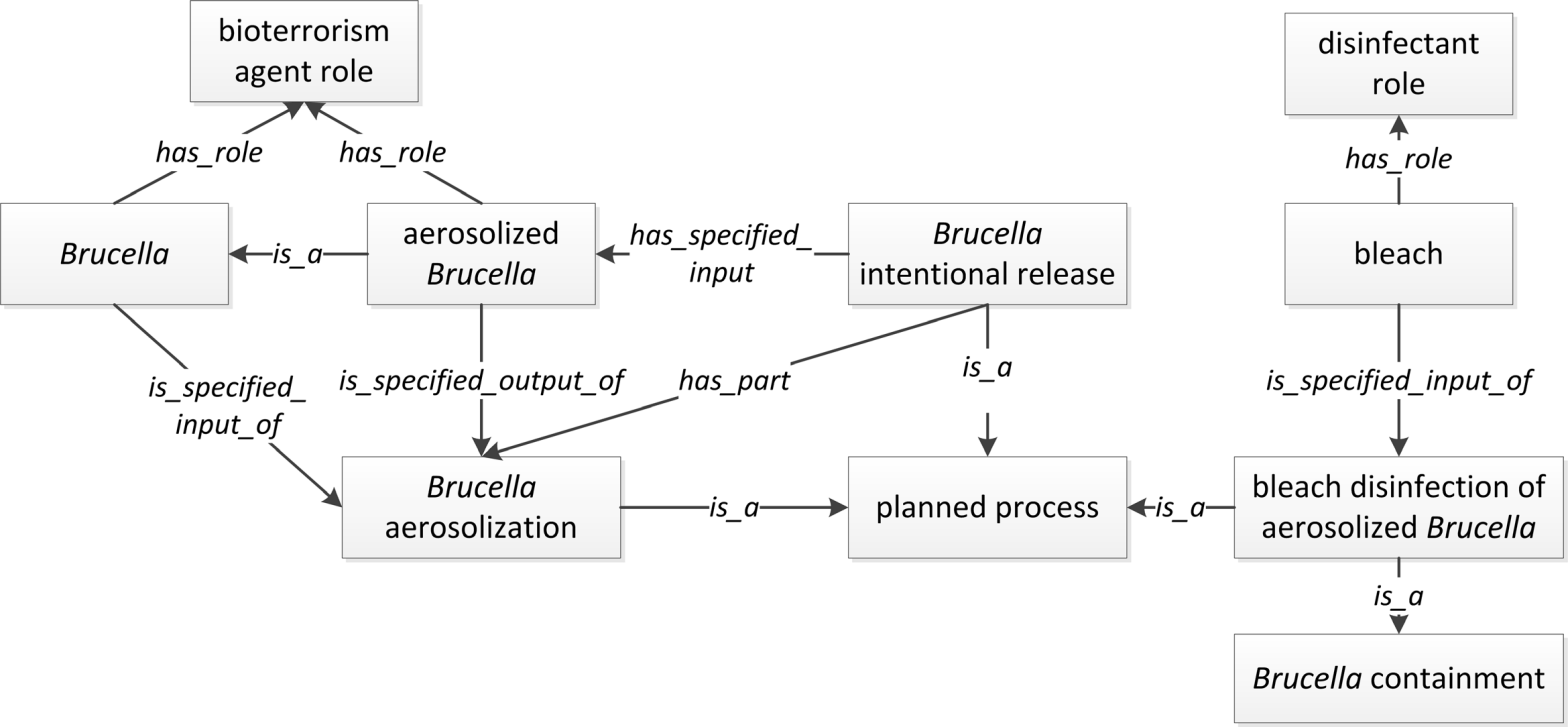
**IDOBRU modeling of *Brucella *intentional release**. The terms inside boxes represent ontology classes. The terms in the middle of arrows are ontology relations.

The intentional release of aerosolized *Brucella *is modeled (Figure 6). Both *Brucella *and *aerosolized Brucella *has the role as a bioterrorism agent. The process of *Brucella intentional release *is a *OBI:planned process*. The *aerosolized Brucella *has been produced by the *Brucella aerosolization *process (a subtype of *OBI:planned process*), which is an integral part of the *Brucella intentional release *process. When *Brucella *is used for the intentional release, *Brucella *is a specified input in the process of *Brucella intentional release*. When a *Brucella *bioterrorism attack occurs, the *bleach disinfection of aerosolized Brucella *process can be initiated for the purpose of disinfection. The *bleach *that bears a *disinfectant role *is a specified input in this process (Figure 6).

### Modeling vaccine prevention of brucellosis in IDOBRU

Vaccination is the most effective means of reducing brucellosis in cattle, sheep and goat [25]. Currently IDOBRU imports 66 *Brucella *vaccines from the Vaccine Ontology (VO; http://www.violinet.org/vaccineontology[21], including four licensed animal *Brucella *vaccines and 62 *Brucella *vaccine candidates that have been proven effective in laboratory animal models [33]. Currently, there is no licensed human brucellosis vaccine. Available animal vaccines may cause disease and are considered unsuitable for use in humans. Nonetheless, a human *Brucella *vaccine is needed to protect the public against human brucellosis and bioterrorism [34].

**Figure 7:**
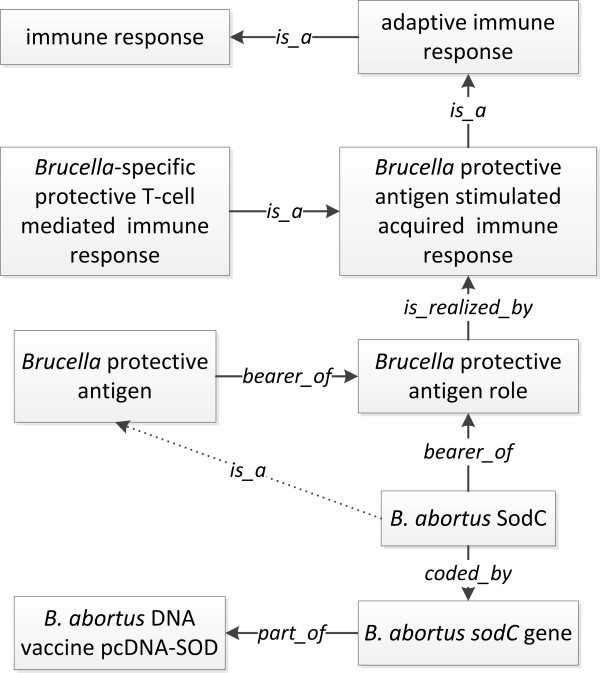
**IDOBRU modeling of a *Brucella *protective antigen and induced immune response**. The terms inside boxes represent ontology classes. The terms in the middle of arrows are ontology relations.

To support rational *Brucella *vaccine design, it is important to understand the protective immunity induced by protective *Brucella *antigens. A protective antigen can stimulate protective adaptive immune response against *Brucella *infection, and is used as an active component of a vaccine. In IDOBRU, this *Brucella*-specific adaptive immune response is a subclass of *GO: adaptive immune response*, which is a *GO:immune response *(Figure 7). For example, *Brucella *Cu/Zn superoxide dismutase (SodC), coded by *Brucella sodC *gene, is a known *Brucella protective antigen *(bearer of the *Brucella protective antigen role*) [35-37]. *Brucella sodC *gene has been used for as part of the *B. abortus *DNA vaccine pcDNA-SOD [38] (Figure 7). Currently IDOBRU has imported 21 *Brucella *protective antigens from VO.

### Modeling treatment of brucellosis in IDOBRU

**Figure 8:**
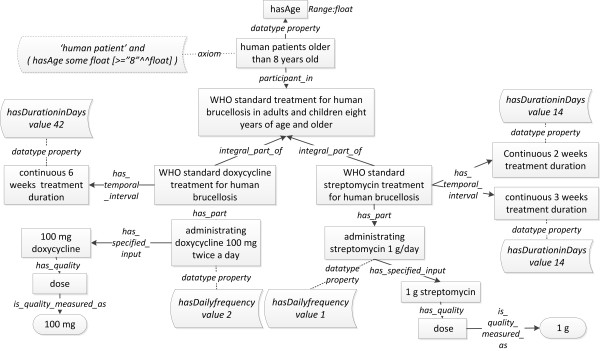
**IDOBRU modeling of brucellosis treatment using a WHO-recommended method**. The two doses (100 mg and 1 g) inside oval boxes are instances. The terms inside square boxes represent ontology classes. The phrase in the dotted box is an axiom. Those phrases inside the other boxes are datatype properties. The terms in the middle of arrows are ontology relations.

Once brucellosis is diagnosed, a treatment will follow. The World Health Organization (WHO) has recommended a standard method for treatment of uncomplicated brucellosis cases in adults and children eight years of age and older: doxycycline 100 mg administrated twice a day for six weeks + streptomycin 1 g daily for two to three weeks [39]. This WHO treatment has been modeled in IDOBRU and described below (Figure 8).

The planned process *WHO standard treatment for human brucellosis in adults and children eight years of age and older *consists of two integral parts: 1) *WHO standard doxycycline treatment for human brucellosis*; 2) *WHO standard streptomycin treatment for human brucellosis*. Using doxycycline treatment as an example, *administrating doxycycline 100 mg twice a day *is part of the example process. Its specified input is the material entity *100 mg doxycycline *with a quality of *dose *measured as 100 mg. A datatype property, *hasDailyfrequency*, has been used for representing the frequency of the administration of doxycycline and its value is 2. The required duration of the whole treatment, is represented by the term *continuous 6 weeks treatment duration*, which is a subclass of *BFO:connected temporal region*. Here we adopted the definition of *connected temporal region *asserted in BFO version 1.1: "A temporal region [span:TemporalRegion] every point of which is mediately or immediately connected with every other point" [40]. Similar to the administration frequency, the 6 weeks' duration is modeled by another datatype property: *'hasDurationinDays value "42"^^integer' *[41]. Since the treatment is limited to patients whose age is greater than 8 years' old, in IDOBRU, the age of a human patient is captured by the datatype property "*hasAge*" with a "float type" restriction. Then, the patient who is suitable for this treatment will be represented by the following constrain as: '*human patient*' *and (hasAge some float [> = "8"^^float])*.

### Ontology reasoning using IDOBRU

Since IDOBRU is developed using the OWL format, OWL-based automated reasoning can be done using reasoning programs within OWL editors or by developing new SPARQL query and reasoning programs. As an example, we designed the following question for querying the ontology: "What *Brucella *virulence factors are also protective antigens?" In IDOBRU, a *Brucella *virulence factor is logically defined as "something that is a *bearer of *some *Brucella virulence factor disposition*". A protective antigen is logically defined as "something that is a *bearer of *some *Brucella protective antigen role*". To answer this question, the following query was performed in the DL Query tab of the OWL editor Protégé 4:

(bearer_of some 'Brucella protective antigen role') and (bearer_of some 'Brucella virulence factor disposition')

**Figure 9:**
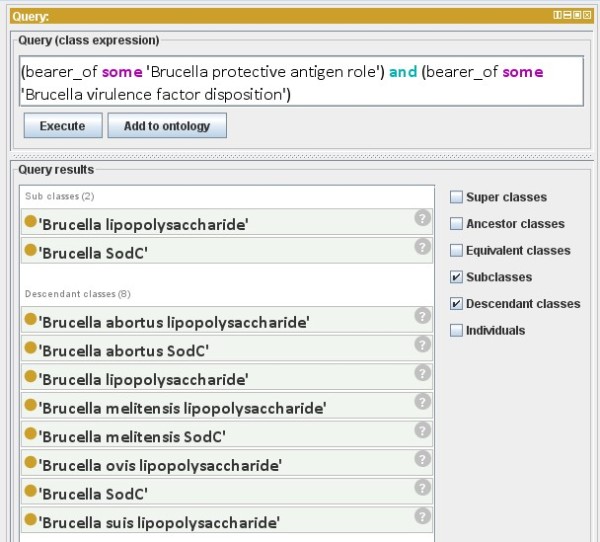
**DL Query window in Protégé 4**. An OWL DL query of *Brucella *virulent factors also being protective antigens using the OWL editor Protégé 4. The query script is shown on the top. The results are displayed at the bottom.

The answers to this query are: *Brucella lipopolysaccharide *and *Brucella sodC *and their six subclasses: *Brucella abortus lipopolysaccharide*, *Brucella abortus SodC*, *Brucella melitensis lipopolysaccharide*, *Brucella melitensis SodC*, *Brucella ovis lipopolysaccharide *and *Brucella suis lipopolysaccharide *(Figure 9).

We have also developed an IDOBRU SPARQL query interface at http://www.phidias.us/bbp/idobru/sparql/index.php. A SPARQL query script has been generated to obtain the same answers by querying the same question (see Additional file 2).

## Discussion

Our approach of developing IDOBRU combines a *top-down *and *bottom-up *realism methodology [42]. In terms of the *top-down *method, we started by making IDO-core as our direct upper ontology, and most of the IDOBRU terms have their respective IDO-core terms as super-classes. The *bottom-up *method is to identify the most specific terms by using scenarios, and then generalize them into higher ontology classes. The prior knowledge of upper ontologies and existing ontologies, especially BFO, RO, IDO, OGMS, VO, and OBI, is essential for the development of IDOBRU. Before a new term is generated, we shall check whether or not this new term and its possible upper level term have existed in other ontologies. Extensive discussion is often needed to achieve a consensus in new term definition.

The limitations of upper ontologies limit ontology development in IDOBRU. For example, the ontological theory of signs and symptoms and their relations to pathological processes has not been well-developed yet. OGMS is in the very early stages of addressing this need [12]. Since it is one of the most important features of the clinical manifestation of human brucellosis, IDOBRU tried to model the undulant fever process (Figure 3). After several rounds of debating and discussing in the community of IDO, finally the agreement has been achieved: (a) the term *elevated temperature *is a *BFO:Quality*, and it is the quality of the *human brucellosis patient*; and (b) the *brucellosis diurnal variation fever process *is a subclass of *OGMS: pathological bodily process*. IDOBRU will evolve while other ontologies make their progress.

IDOBRU also has its own limitations. First, IDOBRU is still at the early stage of its development. As a knowledgebase, the information about brucellosis in IDOBRU is still limited. For example, while we have modeled one PCR assay for brucellosis diagnosis in IDOBRU, many other brucellosis diagnostic assays have been reported in the literature and collected in online resources including the BBP [4]. We also need to model other diagnosis assays including many serological diagnosis methods. Another limitation of IDOBRU exists in its coverage of epidemiological terms. During the course of our IDOBRU development, a large number of epidemiological terms have been generated. Among them, many terms (*e.g*., infectious disease endemic site) are not available in current IDO-core. Other IDO extension ontologies (*e.g*., Influenza Ontology) have also been developing their epidemiological terms. Therefore, closer collaborations among the IDO-core and IDO extension ontologies are needed.

Currently, several IDO extensions are being developed, such as Malaria Ontology [14], Influenza Ontology [13], *Staphylococcus aureus *Ontology, and Tuberculosis Ontology http://infectiousdiseaseontology.org/page/Extensions. Malaria Ontology was reported and published before an IDO-core was released [14]. *Staphylococcus aureus *Ontology was one of the first bacterial extension ontologies developed and has been well integrated with the IDO-core. Both *S. aureus *and *M. tuberculosis *are Gram-positive bacteria. So far *Brucella *has been the only Gram-negative bacterium being studied for the purpose of an IDO extension ontology development. IDOBRU is the first IDO extension ontology that is published after the official release of the IDO-core. It provides a useful real world example of using the IDO-core terms for development of an IDO extension. To our knowledge, IDOBRU is the first ontology that logically models several key topics of an infectious disease, including host infection, zoonotic disease transmission, symptoms, virulence factors and pathogenesis, diagnosis, intentional release relevant to bioterrorism, and treatment.

IDOBRU can be used for several applications. First of all, serving as a knowledgebase of *Brucella *and brucellosis, IDOBRU captures the knowledge extracted from biomedical bench research, clinical practices, and public health. Secondly, owing to the parseable and machine understandable nature of the ontology, IDOBRU supports *Brucella *and brucellosis data exchange, data integration, and automated reasoning. IDOBRU uses BFO, RO and other existing ontologies such as OBI and OGMS. Therefore, the IDOBRU information can easily be integrated with other ontologies and processed with software programs developed based on OBO Foundry Principles. Finally, the development of IDOBRU may serve as an example to model infectious diseases caused by other microbial pathogens. Once many other infectious diseases are modeled using the same framework, it is possible not only to compare different infectious diseases automatically, but also to discover new knowledge in infectious disease domain.

## Conclusions

IDOBRU is a brucellosis-centric ontology and a knowledge-centric platform. IDOBRU can be used as a brucellosis knowledgebase and is applicable for brucellosis data exchange, data integration, and automated reasoning.

## Methods

### IDOBRU editing

IDOBRU was developed using the format of W3C standard Web Ontology Language (OWL2) http://www.w3.org/TR/owl-guide/. For efficient editing of IDOBRU, the Protégé 4.1 OWL ontology editor http://protege.stanford.edu/ was used.

### Existing ontology term import

The whole ontologies of BFO, RO and IDO-core have been imported into IDOBRU using the OWL ontology importing feature. A web server OntoFox http://ontofox.hegroup.org/[15] was used to import external terms from existing ontologies to IDOBRU.

### IDOBRU access and licensing

The latest version of IDOBRU is always available at http://svn.code.sf.net/p/idobru/code/trunk/trunk/src/ontology/brucellosis.owl. In addition, IDOBRU has been deposited in the repositories of NCBO BioPortal http://purl.bioontology.org/ontology/IDOBRU. The IDOBRU source code is freely available under the Apache License 2.0. This licensing allows IDOBRU users to freely distribute and use IDOBRU.

### IDOBRU visualization and term search

To make it convenient for users to browse and search the definitions and usages of IDOBRU terms and their relations, we have developed a user-friendly IDOBRU Browser using the Ontobee linked ontology browser system http://www.ontobee.org/browser/index.php?o=IDOBRU.

### SPARQL query of IDOBRU

The IDOBRU SPARQL query web page is built as a software program compatible with the IDOBRU website http://www.phidias.us/bbp/idobru located in the *Brucella *Bioinformatics Portal (BBP; http://www.phidias.us/bbp) [4,43]. To develop this program, the IDOBRU was stored in a Virtuoso RDF store located in one HP ProLiant DL380 G6 server that runs the Red Hat Linux operating system (Red Hat Enterprise Linux 5 server). PHP scripting language was used for developing the SPARQL web interface. The SPARQL queries provided by a user are implemented through HTTP against the RDF stores.

## List of abbreviations

BFO: Basic Formal Ontology; BBP: *Brucella *Bioinformatics Portal; CHEBI: Chemical Entities of Biological Interest; DL; Description Logic; ELISA: Enzyme-Linked Immunosorbent Assay; GO: Gene Ontology; IAO: Information Artifact Ontology; IDO: Infectious Disease Ontology; IDO-core: Infectious Disease Ontology core; LPS: lipopolysaccharide; NCBI: The National Center for Biotechnology Information; NCBO: The National Center for Biomedical Ontology; OBI: Ontology for Biomedical Investigations; OBO: The Open Biomedical Ontologies; OGMS: Ontology for General Medical Science; OWL: Web Ontology Language; PATRIC: Pathosystems Resource Integration Center; PCR: Polymerase Chain Reaction; PHP: Hypertext Preprocessor; PO: Protein Ontology; RDF: Resource Description Framework; RO: OBO Relation Ontology; ro_proposed: RO proposed version; SPARQL: SPARQL Protocol and RDF Query Language; URI: Uniform Resource Identifier; VIOLIN: Vaccine Investigation and Online Information Network; VO: Vaccine Ontology; WHO: World Health Organization; XML: Extensible Markup Language.

## Competing interests

The authors declare that they have no competing interests.

## Authors' contributions

YL, Primary IDOBRU developer, use case testing, drafting of manuscript. ZX, IDOBRU developer, Webmaster, software programmer, database administrator, and manuscript editing. YH, IDOBRU developer, project design and management, brucellosis domain expert, use case testing, and drafting of manuscript. All authors read and approved the final manuscript.

## Supplementary Material

Additional file 1 **Ontology classes used in the manuscript**. The file includes the detailed information about all ontology classes and relations used in the manuscript. Click here for file

Additional file 2 **SPARQL script**. This SPARQL script is used to query the IDOBRU: "What *Brucella *virulence factors are also protective antigens?". Click here for file
