# Supplementary material for: Brucellosis Ontology (IDOBRU) as an extension of the Infectious Disease Ontology
Source: J Biomed Semantics. 2011 Oct 31;2:9. doi: 10.1186/2041-1480-2-9 (PMC3217896; doi:10.1186/2041-1480-2-9)
Supplement: Additional file 1 — Ontology classes used in the manuscript. The file includes the detailed information about all ontology classes and relations used in the manuscript. [file 2041-1480-2-9-S1.PDF]

Table 1. Ontology classes used in the manuscript (note: instances are not included):

#	Classes	Sources and term IDs	Parent class	Figures
1	*Brucella*	http://purl.org/obo/owl/NCBITaxon#NCBITaxon_234	Alphaproteobacteria	2
2	*Brucella abortus*	http://purl.org/obo/owl/NCBITaxon#NCBITaxon_235	*Brucella*	2
3	*zoonotic disposition*	http://purl.obolibrary.org/obo/IDO_0000453	infectious disposition	2
4	*process of establishing B. abortus infection*	http://purl.obolibrary.org/obo/IDO_0100426	process of establishing an infection	2
5	*cattle brucellosis*	http://purl.obolibrary.org/obo/IDO_0100031	brucellosis	
6	*human brucellosis*	http://purl.obolibrary.org/obo/IDO_0100028	brucellosis	2
7	*human brucellosis pathogen role*	http://purl.obolibrary.org/obo/IDO_0100422	pathogen role	2
8	*cattle brucellosis pathogen role*	http://purl.obolibrary.org/obo/IDO_0100421	pathogen role	2
9	*human brucellosis disease course*	http://purl.obolibrary.org/obo/IDO_0100199	brucellosis disease course	2
10	*cattle brucellosis disease course*	http://purl.obolibrary.org/obo/IDO_0100418	brucellosis disease course	2
11	*Brucella infected cow*	http://purl.obolibrary.org/obo/IDO_0100092	Brucella infected organism	2
12	*Brucella infected human*	http://purl.obolibrary.org/obo/IDO_0100091	Brucella infected organism	2
13	*Brucella-contaminated milk*	http://purl.obolibrary.org/obo/IDO_0100432	material_entity	2
14	*Brucella-contaminated milk producing process*	http://purl.obolibrary.org/obo/IDO_0100478	milk producing process	2
15	*Brucella-contaminated milk producing dispositions*	http://purl.obolibrary.org/obo/IDO_0100130	milk producing disposition	2
16	*drinking function*	http://purl.obolibrary.org/obo/IDO_0100429	function	2
17	*human brucellosis symptom*	http://purl.obolibrary.org/obo/IDO_0100429	brucellosis symptom	3
18	*elevated temperature*	http://purl.obolibrary.org/obo/IDO_0100623	sign	3
19	*normal temperature*	http://purl.obolibrary.org/obo/IDO_0100622	sign	3
20	*body temperature elevation*	http://purl.obolibrary.org/obo/IDO_0100625	bodily process	3
21	*temperature staying normal*	http://purl.obolibrary.org/obo/IDO_0100624	bodily process	3
22	*diurnal variation of temperature*	http://purl.obolibrary.org/obo/IDO_0100022	brucellosis pathological bodily process	3
23	*Brucella virulence factor*	http://purl.obolibrary.org/obo/IDO_0100033	virulence factor	4
24	*Brucella virulence factor disposition*	http://purl.obolibrary.org/obo/IDO_0100116	virulence factor disposition	4
25	*establishment of Brucella intracellular localization*	http://purl.obolibrary.org/obo/IDO_0100614	establishment of localization in cell	4
26	*Brucella's entry into macrophages*	http://purl.obolibrary.org/obo/IDO_0100610	entry of bacterium into host cell	4
27	*process of Brucella's survival in*	http://purl.obolibrary.org/obo/IDO_0100613	biological_process	4

	macrophages			
28	*process of Brucella's replication in macrophages*	http://purl.obolibrary.org/obo/IDO_0100612	biological_process	4
29	*process of establishing Brucella infection in macrophage*	http://purl.obolibrary.org/obo/IDO_0100732	process of establishing Brucella infection	4
30	*Brucella VirB1*	http://purl.obolibrary.org/obo/IDO_0100389	*Brucella* protein	4
31	*Brucella protein virulence factor*	http://purl.obolibrary.org/obo/IDO_0100425	*Brucella* virulence factor	4
32	*Brucellosis diagnosis*	http://purl.obolibrary.org/obo/IDO_0100104	diagnosis	5
33	*PCR assay for detecting Brucella omp-2*	http://purl.obolibrary.org/obo/IDO_0100568	PCR assay for brucellosis	5
34	*omp-2 forward primer*	http://purl.obolibrary.org/obo/IDO_0100067	omp-2 primer	5
35	*omp-2 reverse primer*	http://purl.obolibrary.org/obo/IDO_0100068	omp-2 primer	5
36	*DNA primer sequence data*	http://purl.obolibrary.org/obo/IDO_0100157	DNA sequence data	5
37	*PCR product sequence data*	http://purl.obolibrary.org/obo/IDO_0100603	DNA sequence data	5
38	*omp-2 gene*	http://purl.obolibrary.org/obo/IDO_0100647	Brucella spp. protein coding gene	5
39	*B.abortus genome*	http://purl.obolibrary.org/obo/IDO_0100702	genome	5
40	*omp-2 gene sequence data*	http://purl.obolibrary.org/obo/IDO_0100618	DNA sequence data	5
41	*B.abortus genome sequence data*	http://purl.obolibrary.org/obo/IDO_0100616	DNA sequence data	5
42	*PCR amplification of 193bp region in omp-2 gene*	http://purl.obolibrary.org/obo/IDO_0100107	polymerase chain reaction	5
43	*product of PCR amplification of 193 bp in Brucella omp-2*	http://purl.obolibrary.org/obo/IDO_0100531	PCR product of brucellosis test	5
44	*DNA extracted from brucellosis human patient derived specimen*	http://purl.obolibrary.org/obo/IDO_0100676	brucellosis patient specimen	5
45	*laboratory finding of Brucella omp-2 detection*	http://purl.obolibrary.org/obo/IDO_0100532	laboratory finding	5
46	*brucellosis diagnostic process*	http://purl.obolibrary.org/obo/IDO_0100705	diagnostic process	5
47	*clinical manifestation of brucellosis*	http://purl.obolibrary.org/obo/IDO_0100087	clinical manifestation of brucellosis	5
48	*brucellosis eradication*	http://purl.obolibrary.org/obo/IDO_0100176	eradication of infectious disease	
49	*Brucella accidental release*	http://purl.obolibrary.org/obo/ IDO_0100169	accidental release	
50	*brucellosis free site*	http://purl.obolibrary.org/obo/ IDO_0100536	infectious disease free site	
51	*brucellosis non-endemic site*	http://purl.obolibrary.org/obo/IDO_0100538	infectious disease non-endemic site	
52	*brucellosis surveillance*	http://purl.obolibrary.org/obo/IDO_0100658	infectious disease surveillance	

53	*aerosolized Brucella*	http://purl.obolibrary.org/obo/IDO_0100469	*Brucella*	6
54	*Brucella intentional release*	http://purl.obolibrary.org/obo/IDO_0100169	intentional release	6
55	*Brucella aerosolization*	http://purl.obolibrary.org/obo/IDO_0100470	planned process	6
56	*bioterrorism agent role*	http://purl.obolibrary.org/obo/IDO_0100733	role	6
57	*bleach disinfection of aerosolized Brucella*	http://purl.obolibrary.org/obo/IDO_0100474	disinfection of aerosolized Brucella	6
58	*disinfectant role*	http://purl.obolibrary.org/obo/IDO_0000432	role	6
59	*Brucella protective antigen role*	http://purl.obolibrary.org/obo/IDO_0100119	protective antigen role	7
60	*Brucella-specific protective T-cell mediated immune response*	http://purl.obolibrary.org/obo/IDO_010740	*Brucella* protective antigen stimulated acquired immune response	7
61	*Brucella protective antigen stimulated acquired immune response*	http://purl.obolibrary.org/obo/IDO_010740	adaptive immune response	7
62	*Brucella abortus SodC*	http://purl.obolibrary.org/obo/IDO_010734	*Brucella* protein	7
63	*B.abortus sodC gene*	http://purl.obolibrary.org/obo/IDO_010736	*Brucella* protein coding gene	7
64	*B.abortus DNA vaccine pcDNA-SOD*	http://purl.obolibrary.org/obo/VO_0000018	Brucella abortus vaccine	7
65	*WHO standard treatment for human brucellosis in adults and children eight years of age and older*	http://purl.obolibrary.org/obo/IDO_0100185	WHO recommended human brucellosis treatment	8
66	*WHO standard doxycycline treatment for human brucellosis*	http://purl.obolibrary.org/obo/IDO_0100527	brucellosis treatment	8
67	*WHO standard streptomycin treatment for human brucellosis*	http://purl.obolibrary.org/obo/IDO_0100528	brucellosis treatment	8
68	*doxycycline*	http://purl.obolibrary.org/obo/IDO_0100113	antibiotic	8
69	*continuous treatment duration*	http://purl.obolibrary.org/obo/IDO_0100620	connected temporal region	8
70	*administrating doxycycline 100mg twice a day*	http://purl.obolibrary.org/obo/IDO_0100734	treatment	8
71	*administrating streptomycin 1g a day*	http://purl.obolibrary.org/obo/IDO_0100735	treatment	8

Table 2. Relations used in the manuscript:

#	Property terms	Sources and term IDs	Figures
1	bearer_of	http://purl.org/obo/owl/OBO_REL#bearer_of	2,7
2	realized_by	http://purl.org/obo/owl/OBO_REL#realized_by	2,4,7
3	role_of	http://www.obofoundry.org/ro/ro.owl#role_of	2
4	has_specified_output	http://purl.obolibrary.org/obo/OBI_0000299	2,5
5	is_specified_input_of	http://purl.obolibrary.org/obo/OBI_0000295	2,5,6
6	has_function	http://purl.obolibrary.org/obo/OBI_0000306	2
7	has_quality	http://purl.org/obo/owl/OBO_REL#has_quality	3,8
8	has_part	http://www.obofoundry.org/ro/ro.owl#has_part	3,4,5,6,8
9	has_scattered_temporal_region	http://purl.obolibrary.org/obo/IDO_0100626	3
10	participates_in	http://www.obofoundry.org/ro/ro.owl#participates_in	4,8
11	has_disposition	http://purl.org/obo/owl/OBO_REL#has_disposition	4
12	has_role	http://purl.obolibrary.org/obo/OBI_0000316	6
13	has_specified_input	http://purl.obolibrary.org/obo/OBI_0000293	6,8
14	is_about	http://purl.obolibrary.org/obo/IAO_0000136	5
15	inheres_in	http://purl.org/obo/owl/OBO_REL#inheres_in	5
16	achieves_planned_objective	http://purl.obolibrary.org/obo/OBI_0000417	5
17	part_of	http://www.obofoundry.org/ro/ro.owl#part_of	5,7
18	integral_part_of	http://www.obofoundry.org/ro/ro.owl#integral_part_of	8
19	coded_by	http://purl.obolibrary.org/obo/IDO_0100541	7
20	has_temporal_interval	http://purl.obolibrary.org/obo/IDO_0100621	8
