# Supplementary material for: Brucellosis Ontology (IDOBRU) as an extension of the Infectious Disease Ontology
Source: J Biomed Semantics. 2011 Oct 31;2:9. doi: 10.1186/2041-1480-2-9 (PMC3217896; doi:10.1186/2041-1480-2-9)
Supplement: Additional file 2 — SPARQL script. This SPARQL script is used to query the IDOBRU: "What Brucella virulence factors are also protective antigens?". [file 2041-1480-2-9-S2.PDF]

The following SPARQL script is used to query the IDOBRU: “What Brucella virulence factors are also protective antigens?”

```
select distinct ?s, ?l

from <http://purl.obolibrary.org/obo/ido/brucellosis.owl>

 where
{
{
?s rdfs:label ?l .
?s rdfs:subClassOf ?s1.

?s1 owl:onProperty <http://purl.org/obo/owl/OBO_REL#bearer_of>.
?s1 owl:someValuesFrom <http://purl.obolibrary.org/obo/IDO_0100119>.


?s rdfs:subClassOf ?s2.
?s2 owl:onProperty <http://purl.org/obo/owl/OBO_REL#bearer_of>.
?s2 owl:someValuesFrom <http://purl.obolibrary.org/obo/IDO_0100116>

}

UNION
 {
?s rdfs:label ?l .
?s rdfs:subClassOf ?s0.
?s0 rdfs:subClassOf ?s1.

?s1 owl:onProperty <http://purl.org/obo/owl/OBO_REL#bearer_of>.
?s1 owl:someValuesFrom <http://purl.obolibrary.org/obo/IDO_0100119>.


?s0 rdfs:subClassOf ?s2.
?s2 owl:onProperty <http://purl.org/obo/owl/OBO_REL#bearer_of>.
?s2 owl:someValuesFrom <http://purl.obolibrary.org/obo/IDO_0100116>

}

}
```
